# Motion, identity and the bias toward agency

**DOI:** 10.3389/fnhum.2014.00597

**Published:** 2014-08-21

**Authors:** Chris Fields

**Affiliations:** New Mexico State UniversityUSA (Retired)

**Keywords:** analogy, causal reasoning, infant cognition, mirror neuron system, structure mapping, systemizing

## Abstract

The well-documented human bias toward agency as a cause and therefore an explanation of observed events is typically attributed to evolutionary selection for a “social brain”. Based on a review of developmental and adult behavioral and neurocognitive data, it is argued that the bias toward agency is a result of the default human solution, developed during infancy, to the computational requirements of object re-identification over gaps in observation of more than a few seconds. If this model is correct, overriding the bias toward agency to construct mechanistic explanations of observed events requires structure-mapping inferences, implemented by the pre-motor action planning system, that replace agents with mechanisms as causes of unobserved changes in contextual or featural properties of objects. Experiments that would test this model are discussed.

## Introduction

That human beings exhibit a bias, beginning in infancy, toward explaining events in terms of agency is well documented (reviewed by Bloom, 2007; Boyer and Bergstrom, 2008; Rosset, 2008; Waytz et al., 2010). The classic experiments of Heider and Simmel (1944) showing that subjects readily attribute agency to animated geometrical shapes, which have been repeated and elaborated upon in the years following (reviewed by Scholl and Tremoulet, 2000; Scholl and Gao, 2013), remain one of the most striking demonstrations of this bias toward agentive explanation. Gao et al. (2010), for example, showed not only that adult subjects “irresistibly” attribute both agency and malign intent to displays of “V” shapes moving in particular ways, but also that this spurious attribution of agency disrupts performance on a multiple-object tracking task. The human bias toward agentive explanation is not, of course, restricted to the laboratory. Evidence from multiple cultural groups increasingly indicates that adults regularly explain events by appeal to a combination of supernatural agentive causes and natural, non-agentive causes (Legare et al., 2012). That supernatural and teleological causes tend to be invoked preferentially under conditions involving time limitations (e.g., Gelman and Legare, 2011) or stress (e.g., Paul, 2009) suggests that they represent a default strategy and hence a cognitive bias; indeed Kelemen et al. (2013) have recently shown that even professional physical scientists readily accept teleological explanations of natural phenomena when stressed with time limitations. While technological culture by itself might be expected to reduce the frequency of explanatory appeals to supernatural agency in particular, the high level of religiosity in the United States (Paul, 2009) indicates otherwise. Indeed, technological culture both promulgates and benefits from the bias toward agency in areas ranging from automobile esthetics to the ubiquitous and increasingly psychologically-sophisticated use of the “agent metaphor” in user-interface design (e.g., Schiaffino and Amandi, 2004).

What remains less clear is the source of this bias toward agentive explanation. While robust traditions of psychological, sociological and cultural analyses have focused on the emergence and maintenance of the religious manifestation of the bias toward agency in communities of adults (see reviews by Main, Davie and Bowie, respectively, in Segal, 2006), research over the last two decades increasingly indicates that the bias toward agency develops in early infancy and depends only on non-specific experience (reviewed by Luo and Baillargeon, 2010; Bremner, 2011; Csibra and Gergely, 2012; Rottman and Kelemen, 2012; see Vaden and Woolley, 2011 for evidence that the religious manifestation of the bias does depend on specific religious experience). Employing cross-cultural data, Kelemen (2012), for example, discounts parental, social and broader cultural influences as explanations of the “promiscuous teleology” of children in favor of a cognitive developmental explanation based on the possession by infants of “intentional agent” as a fundamental category and their tendency to interpret all observed events as caused, directly or indirectly, by such agents. The origin of the category “intentional agent” and the tendency of infants, children and even adults to employ it ubiquitously is often explained in turn by appeal to the “social brain hypothesis” that human cognitive architecture is an evolutionary adaptation to a selective environment in which competing individuals were and still are forced to cooperate within cohesive social groups (reviewed by Adolphs, 2003, 2009; Dunbar, 2003; Dunbar and Shultz, 2007). According to this hypothesis, the agentive actions of conspecifics, whether friends or foes, largely defined the selective environment of hominid evolution; the over-application of agentive explanations outside the social sphere is an unsurprising consequence of the tendency of selective pressures to favor false positives from risk detectors over false negatives. While this evolutionary account explains why a bias toward agency might be expected in human populations, however, it does not explain either how the bias is implemented or its developmental time course.

As noted, the bias toward agency first becomes apparent in early infancy. While infants are able to categorize observed objects as “inanimate” and even as “self-propelled inanimate” (Luo et al., 2009; Rakison and Yermolayeva, 2010), infant recognition of non-agentive causation appears to be limited to directly-observed, contact-dependent transfers of mechanical force (Spelke, 1994). Changes in behavior that do not result from observed contact forces, such as autonomous changes in direction of motion that result in circumvention of an obstacle, indicate agency. Infants are surprised by self-propelled inanimate objects and often (see below) categorize them as “agents”. Considerable evidence indicates that the recognition of agency is implemented, at least in part, by the mirror neuron system (MNS; reviewed by Cattaneo and Rizzolatti, 2009; Rizzolatti and Sinigaglia, 2010; Casile et al., 2011). As the origin, development, functions and anatomical extent of the MNS, the extent to which mirror neurons as originally defined (di Pellegrino et al., 1992) contribute to perception-action “mirroring” as a functional outcome, and the extent to which the association of perceived actions with heterologous motor representations can be considered a “mirror” function are all subject to considerable debate (e.g., Gallese et al., 2011; Cook et al., 2014), here the term “MNS” is used broadly to refer to components of the visuo-motor system that produce specific motor excitations in response to specific visual perceptions and hence implement mirroring—again broadly conceived—as a function. Some recent evidence suggests that this mirroring function, and hence an MNS, is sufficiently developed at birth to allow the discrimination between erect point-light walkers and inverted ones (Simion et al., 2008; Bardi et al., 2011; see Pavlova, 2012 for a review of contrasting findings). Early involvement of the MNS in the recognition of agency is particularly indicated by the correlation between infant abilities to imitate actions—in some cases, only after appropriate training—and to recognize those same actions as goal-directed and hence intentional when they are performed by other entities categorized as agents (reviewed by Woodward et al., 2009; Marshall and Meltzoff, 2011). The parallel development of agency detection and MNS capabilities in infants, together with the very limited and specific infant abilities to recognize non-agentive causes, suggests that agency can be regarded as an experience-dependent but nonetheless architecturally-specified default representation of causation in infancy. The question of why the bias toward agency survives into adulthood can, therefore, be viewed as the question of why this infant default should survive post-infancy experience, and in particular, why it should survive post-infancy experience in highly-technological cultural settings in which self-propelled or otherwise self-powered artifacts are both ubiquitous and available for exploratory investigation, and in which explicit educational instruction in mechanistic thinking is nearly universal. Recent evidence that adults cross-culturally are even more biased toward supernatural agency, in particular, as an explanation than are children (Woolley et al., 2011) makes this question even more urgent. Assuming that the bias toward agency is an evolutionary outcome as postulated by the social brain hypothesis, what is it about the implementation of this outcome that makes it so refractory, both to experience and to consciously-accessible explicit knowledge? At least some children exposed to self- or remotely-powered artifacts develop an understanding that internal or remote mechanisms can cause complex behaviors during the preschool years (Kushnir and Gopnik, 2007; Sobel et al., 2007; Sobel and Buchanan, 2009; Buchanan and Sobel, 2011), a period during which “folk physics” capabilities are rapidly developing (Karmiloff-Smith, 1995). Why does the attribution of agency to, for example, animated geometrical shapes survive this transition to a post-infancy understanding of mechanism-driven causality?

The present paper proposes that the infant bias toward agency is maintained, at least in part, by the computational requirements of re-identifying individual objects as the same individuals across changes in features and context. As emphasized by Baillargeon (2008), the human cognitive architecture appears to implement, from birth, a “principle of persistence” according to which objects “not only exist continuously and remain cohesive, they also retain their individual properties” (p. 3) in the absence of identifiable causal influences. Hence infants are surprised, for example, when an object has disappeared or its position has changed following a brief occlusion. As infants mature, they are increasingly capable of re-identifying objects after substantial “occlusions”—for example, after waking up the next morning—in a way that is robust against differences in both object locations and features. Re-identifying objects as persistent individuals across substantial gaps in observation during which context-changing motions and/or featural changes have occurred requires attributing an unobserved cause to the observed differences in context or features (reviewed by Rips et al., 2006; Scholl, 2007; Fields, 2012a). If agency is the default cause of motion and feature change available in infancy and early childhood, unobserved agentive causes can be expected to be associated with every individual object on every occasion of its re-identification in a different context or with altered features. Attributing a mechanical cause to differences in the features or context of an object re-identified following a gap in observation requires overriding this default within the object re-identification system. It is suggested that mechanical causes become available to the object re-identification system via structure mapping inferences, the kind of inferences that implement analogies (Gentner, 1983; Markman and Gentner, 2001), and that they become available only following appropriate experience. Evidence from a variety of sources suggests that these structure-mapping inferences are implemented by the pre-motor action-planning system (Schubotz, 2007; Bubic et al., 2010; Fields, 2011a, 2012b). If this model of object re-identification is correct, agentive explanations remain the default into adulthood unless they are replaced, via this structure-mapping process, by mechanical explanations in the functional context of the object re-identification system.

The sections that follow review the evidence supporting this model and discuss experiments that would further test it. The next section, “Background” briefly reviews the past decade of experimental work on visual indicators of agency during infancy, infant abilities to recognize and imitate actions performed by entities identified as agents, and pre-school abilities to understand mechanism-driven causation. The latter studies show that while agentive explanations remain a default for pre-schoolers, they can override this default in at least some contexts to produce explanations that appeal to hidden mechanisms as causes. The third section, “The Role of Causal Reasoning in Tracking Object Identity Over Time” reviews evidence that representations of unobserved causal processes encoded by the pre-motor action-planning system play an obligate role in re-identifying objects across gaps in observation of more than a few seconds. If all unobserved causal processes are agentive, their role in object re-identification over time renders object identity itself dependent on agency. The fourth section, “Mechanism-Driven Causation as An Obligate Analog of Agency” reviews evidence that structure mapping inferences are required to replace representations of internal or external agents with representations of unobserved mechanisms as the causes of changes in object locations or features across gaps in observation. As such inferences require cognitive resources, the explanation of observed changes by appeal to unobserved mechanisms is intrinsically more resource-intensive than their explanation by appeals to unknown agents. The fifth section, “Overriding the Bias Toward Agency” summarizes the resulting model of the bias toward agency, its maintenance into adulthood, and the kinds of experiences required to override the bias; it also discusses experiments that would further test the model. The paper concludes by briefly exploring the link between “social brains” and the ability to re-identify objects over time that the model implies. Such an ability is clearly required by any social organization that requires its members to recognize individual conspecifics over extended time. The model thus suggests that the social utility of recognized individuality, not just requirements for group cohesion or cooperation in the face of similarly-organized competitors, drives the evolution of social brains.

## Background

### Indicators of agency in infancy

“Agent” appears to be a fundamental, and quite possibly innate, ontological category for human infants (Luo and Baillargeon, 2010; Bremner, 2011; Csibra and Gergely, 2012; Rottman and Kelemen, 2012). One can ask, therefore, what perceptual cues indicate agency to infants, and if or how these indicators change over the course of infancy. In particular, one can ask what indicators are sufficient, either individually or collectively, as indicators of agency to infants, and whether any of these sufficient indicators are also necessary.

Infants orient from birth toward human faces (reviewed by Simion et al., 2011). One can hypothesize, therefore, that having a human or human-like face is a sufficient indicator of agency for infants. Kamawari et al. (2005) tested this hypothesis by comparing the responses of 6.5 month-old infants to three displays showing candidate “agents” approaching a stationary target—a stepped block—via either a straight path or a path containing a detour. Unnecessary detours by either a human actor or a robot with a human-like face were surprising to infants, as measured by increased looking time, while an unnecessary detour by a featureless rectangular block elicited no surprise. A moving object with a human-like face, therefore, appears sufficient to indicate agency to 6.5-month-old infants. A subsequent experiment (Csibra, 2008) modeled closely on that of Kamawari et al. showed, however, that a human-like face is not necessary. In this experiment, the responses of 6.5-month-old infants to an unnecessary detour by a featureless rectangular block were compared with the independent variable being whether the infants had previously watched the block detour around a visible obstacle using only one path—a condition that replicated the design of Kamawari et al. (2005)—or using two paths of equal efficiency. Infants who had previously observed the block follow multiple paths to the target were surprised by the unnecessary detour, suggesting an attribution of agency to the moving block. This result is consistent with other studies (Biro and Leslie, 2007; Luo, 2011) that demonstrate equifinal variations of actions by self-propelled objects as an indicator of agency in infants as young as 3 months.

These results, which rely on a visual perception task and therefore provide information about visual information-processing pathways, indicate that while feature information—a human-like face—may be sufficient as an indicator of agency, trajectory information alone is also sufficient. Whether trajectory information is necessary has not been determined; ethical experimental protocols cannot test, for example, whether having a human-like face would indicate agency to young infants even if entities with human-like faces were never observed to move. Even with this proviso, however, a significant fact about architecture can be inferred: while activations of the ventral “what” visual pathway that processes feature information can contribute to the identification of agents by infants, activations of the dorsal “where” visual pathway that processes trajectory information can identify agents in the absence of agent-specific feature information. This dominance of dorsal trajectory recognition over ventral feature recognition for agency detection makes sense on evolutionary grounds. In the ancestral human niche, most self-propelled objects were agents: humans or other animals. If agents are more likely to be either helpful or dangerous than non-agents, being able to identify them using only the relatively-fast, feature-independent dorsal stream is advantageous. Dorsal-stream dominance is also familiar: trajectory information dominates feature information in determining what is represented, at the object-file level, as a persistent entity within a visual scene (reviewed by Treisman, 2006; Scholl, 2007; Flombaum et al., 2008; Fields, 2011b). Hence sufficient indicators of agency are present in the object file, the initial visual-system representation on which all downstream processing acts, in feature-independent form whenever a *moving* agent is detected. From this it can be inferred that excitations of superior temporal sulcus (STS) are components of the initial visual representation of an observed agent (Rizzolatti and Matelli, 2003; Nassi and Callaway, 2009). The initial visual representations of non-agents—passive objects that move only when acted upon by contact forces or gravity, and self-propelled objects that follow invariant, typically linear trajectories—can similarly be expected to involve excitations of medial temporal gyrus (MTG).

As infants continue to mature from 6 months to the onset of communicative language use at 18–24 months, their abilities to represent features of objects and hence distinguish between individual agents or categories of agents progressively improve (Baillargeon et al., 2011, 2012). As discussed below, their abilities to recognize and imitate actions also improve. Available evidence does not, however, suggest that either the perceptual indicators of agency or the variety of objects to which agency may be attributed change during this period; merely being self-propelled, for example, is still demonstrably insufficient as an indicator of agency at 18 months (Cicchino et al., 2011). Indeed, adult tendencies to attribute agency to animated geometric shapes using the same trajectory indicators used by infants (Scholl and Tremoulet, 2000) suggest that these indicators remain stable across the lifespan.

The experiments reviewed above would be uninterpretable without the assumption that the infants involved, starting at the youngest ages tested (i.e., 3 months), are *aware* of both the moving objects and their motion. Nothing in either the experiments or the inferences drawn from them, however, implies or even suggests that the infants involved are aware of their categorization of the objects involved as agents. As Whitehead (2001) has emphasized, the conflation of awareness with awareness of awareness is a common cause of confusion and over-statement in cognitive science; a similar confusion must be avoided here. While it is justified to regard these infants as possessing a procedurally-implemented perceptual *category* “agent”, it is unjustified—at least on the basis of the results reviewed here—to regard them as possessing a consciously accessible *concept* “agent” prior to their acquisition of theory-of-mind (ToM) language in the late preschool years (Saxe et al., 2004).

### Action recognition and imitation in infancy

Mirror system function in infants has been examined at the behavioral level using both looking time and imitation measures. As noted, even 2-day-old infants appear to be more interested, as measured by looking time, in an upright point-light walker display than an inverted display, even when the display shows a hen walking as opposed to a human (Simion et al., 2008; Bardi et al., 2011). Similar experiments suggest that the direction of motion of a human point-light walker display, which remains stationary as if on a treadmill, is detectable by 6-month-old infants (Kuhlmeier et al., 2010). The interpretation of looking-time results is not completely straightforward (for discussions see Aslin, 2007; Turk-Browne et al., 2008) and may be particularly problematic in the case of point-light walkers, which are neither connected nor bounded and thus violate two of the key “principles” of persistent object identification (Baillargeon, 2008; Flombaum et al., 2008). However, the apparent ability of even the youngest infants to “notice” point-light walkers as interesting and to extract useful information from such displays suggests that infants are able to construct object files that capture coherent motion at a high level of abstraction (Fields, 2011b).

While the role of MNS in action imitation remains controversial as noted (Gallese et al., 2011; Cook et al., 2014), considerable evidence now indicates that action-imitation abilities are strongly dependent on prior experience with action performance. For example, 3-month-old infants provided with and trained in the use of sticky mittens that enabled them to “grasp” objects even before the typical onset of coordinated grasping were able to imitate observed grasping actions by an adult wearing similar mittens, while infants provided with mittens but not trained in their use were unable to perform or imitate grasping (Woodward, 2009). The correlation between the ability to imitate an action and prior spontaneous or trained performance of that action continues through later childhood (reviewed by Del Giudice et al., 2009; Woodward et al., 2009; Ray and Heyes, 2010) and into adulthood (Heyes, 2012). Infant imitation is not restricted to human or even animate actors; even 9-month-old infants will imitate, to the extent possible given their behavioral repertoires, the actions of mechanical devices provided that they perceive such devices as agents (Boyer et al., 2011). Such imitation of mechanical devices becomes commonplace in the preschool years, when children frequently imitate the sounds and motions of cars, airplanes, or other machines—often accompanied by announcements such as “I’m an airplane”—in unstructured play.

As the defining function of the MNS is to excite motor representations of actions in response to observations of actions, it is natural to hypothesize that MNS activity is centrally involved in planning the actions that implement imitation in infants and young children. Observations of presumptive MNS activity in infants using high-resolution EEG recordings support this hypothesis (Southgate et al., 2009; Kanakogi and Itakura, 2011; Nyström et al., 2011; Virji-Babul et al., 2012; reviewed by Marshall and Meltzoff, 2011). The plasticity of the MNS, in particular its ability to respond in an experience-dependent manner to non-biological as well as biological motions (Schubotz and von Cramon, 2004; Engel et al., 2007; reviewed by Catmur et al., 2007; Heyes, 2010), is consistent with MNS involvement in the imitation of mechanical devices as well as human or other animate agents.

While imitation ability is sometimes characterized in terms of “intention reading” (e.g., Woodward et al., 2009; Luo and Baillargeon, 2010), it is important, as noted earlier, to avoid the inference that infants are reflectively aware of either the goals of observed actions or the intentions of the agents who perform them (Paulus, 2012a), or that infants “rationally”—again in any sense requiring reflective awareness—choose imitative actions that meet mechanical criteria of efficiency (Paulus, 2012b). Activity in the MNS does not entail awareness of what the MNS is computing; the results reviewed are consistent with the view that infants are aware of the actor and action being imitated, and are aware of their own imitative actions, but are not aware that their actions are imitative. They are, similarly, consistent with the view that infants *categorize* some observed actions as goal-directed and hence intentional, but lack any consciously-accessible *concepts* of goal-directedness or intentionality prior to the development of ToM language.

### Development of mechanism-based causal reasoning in childhood

Pre-school children in industrialized cultures—at least within the affluent demographic primarily studied by developmental cognitive psychologists (Henrich et al., 2010)—live in environments in which self-propelled devices capable of variable and sometimes complex behaviors are both common and available for manipulation and exploration; many such devices are, indeed, toys intended for use by pre-schoolers. Children as young as 3 years old are sufficiently familiar with batteries, for example, to understand that their presence is necessary to make some devices display expected behaviors (Buchanan and Sobel, 2011). From the perspective of infant object categorization, this is a striking ability: a battery is a passive, non-self-propelled object, a battery-less mechanical toy is a passive, non-self-propelled object, but a mechanical toy with batteries installed may be self-propelled and display other behaviors indicative of agency, and may indeed be regarded by the child as an agent. How pre-schoolers represent such categorization conflicts, and whether they resolve them in the context of reflectively-accessible “mental models” (Gentner, 2002) of devices such as mechanical toys remains to be investigated. Ordinary observations, however, are sufficient to show that children find the resolution of such conflicts enjoyable and rewarding, just as adults find understanding “how things work” enjoyable and rewarding (reviewed by Fields, 2011c).

By 4 years old, children are able to identify unfamiliar internal parts of objects as conferring causal power in the way that batteries do (Sobel et al., 2007), although 3-year-olds lack this more general ability. Four-year-olds are also able to recognize that visible connections from one object to another—such as wires—are able to transfer causal power even though they do not move and hence do not transfer mechanical force; again, 3-year-olds lack this ability (Buchanan and Sobel, 2011). Between 4 and 5 years old, children develop an understanding of non-contact causation that operates at a distance, in the way a TV remote control does (Kushnir and Gopnik, 2007; Sobel and Buchanan, 2009). In all of these cases, the causally-efficacious part employed in the experiments—whether an unfamiliar internal component, a wire-like connection, or a colored block capable of remotely activating a “blicket” detector—was otherwise a passive, non-self-propelled object, as was the object that the causally-efficacious part activated. Hence all of these experiments required children to resolve categorization conflicts involving passive, non-self-propelled objects acting in agent-like ways when appropriately associated.

It is worth emphasizing how far from contact forces and rigid-body mechanical motions—the core domain of folk physics learned from infancy by observation and exploratory manipulation (McCloskey, 1983; Karmiloff-Smith, 1995)—these pre-school abilities to recognize otherwise passive parts as causally efficacious go. The pushes and pulls of rigid-body mechanical forces are readily understandable in terms of somatosensory and proprioceptive sensations and felt muscular effort; such non-verbal kinematic and dynamic understanding enables tool improvisation using rigid bodies not just in humans, but in many other species (Fields, 2011a). Neither somatosensory and proprioceptive sensations nor felt muscular effort, however, is sufficient to understand that a battery can make a mechanical toy go, or that a remote control can turn on a television. While an appreciation of folk physics confers considerable predictive power, the ability to attribute internal structure or remote causal powers to otherwise passive objects is the requisite first step toward the kind of analytical thinking that underlies reverse engineering and other forms of diagnostic reasoning as well as all theoretical sciences concerned with *how things work* as opposed to merely *what things do*. Baron-Cohen (2002, 2008) has termed this particular kind of analytical thinking “systemizing” to emphasize that it involves viewing complex objects as systems of interacting components. The experiments of Gopnik, Sobel and their colleagues demonstrate that robust systemizing abilities begin to develop, given appropriate experience, between 3 and 5 years of age.

Systemizing is not the only ability that children begin to develop between 3 and 5 years of age: this is also the time when they begin to develop robust ToM abilities, in particular, the ability to attribute explicit, linguistically-reportable mental-state contents to others (reviewed by Saxe et al., 2004). Sobel and Munro (2009) have provided evidence that these co-developing abilities are non-trivially related by showing that 3-year-olds could recognize an object with hidden causal power if they construed it mentalistically as “liked by” a target object, even though they were unable to identify such objects as having hidden causal power under a mechanistic construal. Indeed throughout this age range, as well as after, children display a marked preference for mentalistic explanations, including teleological explanations, over mechanistic explanations (reviewed by Kelemen, 2004); their new-found ability to understand causal mechanisms sometimes overrides but does not replace the bias toward agency in their understanding of the world. It will be argued in the Section, Mechanism-Driven Causation as an Obligate Analog of Agency below that the co-development of systemizing and ToM abilities may be the key to understanding *how* the capability for systemizing develops during pre-school childhood.

## The role of causal reasoning in tracking object identity over time

With the above background, the question posed in the introduction can be formulated more precisely: if pre-school children develop an understanding of mechanism-driven causation—possibly only an implicit, unverbalized understanding—that enables them to grasp the fact that passive inanimate objects such as batteries or wires can cause otherwise passive inanimate objects to behave in complex, unpredictable ways, or to grasp the fact that such causal powers can act over a distance without mechanical contact, why do they still exhibit a general preference for causal explanations that appeal to agency? Why, in particular, does this new understanding not lead to the replacement of agentive causation by mechanical causation as the default explanation of the behavior of inanimate objects? One possible answer to this question is that explanations that appeal to agency are *simply easier to construct* than explanations that appeal to internal, remote, or otherwise non-obvious mechanisms. It is often claimed, for example, that systemizing is a poor strategy for understanding human behavior because human behavior is complex and unpredictable (e.g., Baron-Cohen, 2002). Implicit in this claim is the idea that it is somehow easier to postulate an unknown intention than it is to postulate an unknown mechanism, and perhaps easier as well to imagine a situation-specific intention to explain an unexpected or unusual occurrence that it is to imagine a situation-specific mechanism. The results of Kelemen (2004, 2012) suggest that young children find it easier to construct teleological explanations than non-teleological ones; the experiments of Kelemen et al. (2013) with Ph.D.-level scientists and humanities scholars as well as the prominent cross-cultural role of adult religion as an explanation of “inexplicable events” suggests that the same is true of adults. Hence our motivating question can also be posed as: why should it be *easier—*less resource-intensive, or less architecturally complex—for the human neurocognitive system to construct agent-based rather than mechanism-based explanations of events? What is it about the representation of causes that makes representing an unobserved agentive cause easier than representing an unknown mechanical cause? The evolutionary considerations underlying the social brain hypothesis suggest that an asymmetry in the representation of agentive vs. mechanical causes may be advantageous, but they shed no light on this question of implementation.

The primary hypothesis of the present paper is that a pre-motor representation of agency as a cause of both motion and featural change plays a critical role, throughout infancy and into early childhood, in the developing ability to represent objects as persistent through time, and therefore in the developing ability to *re-identify* objects as individuals across increasingly large gaps in observation. A corollary of this hypothesis is that the ability to represent an internal or remote mechanism—particularly an unobserved mechanism—as a cause of either motion and featural change is derivative, by structure mapping inferences, from the ability to represent agents as causes. If this hypothesis is correct, the human bias toward agency survives from infancy into adulthood because mechanism-based causal reasoning is in fact harder than agent-based causal reasoning: it requires additional inferential steps and therefore additional neurocognitive capabilities and resources. The remainder of this section reviews evidence supporting the hypothesis that pre-motor representations of causality enable object re-identification across gaps in observation. The next section reviews evidence that mechanism-driven causation is an obligate analog of agency-driven causation. The final section discusses experimental designs to further test the resulting model of object re-identification.

Alert infants are not indifferent to changes occurring in their environments; however, the changes to which they are sensitive are highly dependent on both age and experience. Four and a half month-old infants, for example, are initially unable to segregate displays of unfamiliar stationary solid objects into distinct, bounded entities, but with appropriate experience with objects similar to those displayed can do so (Needham et al., 2005). Spelke (1994) synthesized results from early experiments with infant object segregation and re-identification to propose that infants employ innate “principles” of cohesion and continuity of motion to segregate objects, and an innate principle of physical contact for the transmission of mechanical forces. This latter principle applies in particular to inanimate objects, which are typically not self-propelled and hence move only when acted upon by an agent; it applies, moreover, to physical contact that has been *observed*. With results from many subsequent studies, Baillargeon (2008) generalized from the principles of cohesion and continuity of motion to propose that infants employ an innate principle of persistence for both objects and their attended properties, with the explicit proviso that the types of properties taken to be persistent depend on age and experience (see also Baillargeon et al., 2011, 2012). Statements that such principles constitute a fundamental form of “knowledge” (Spelke, 1994) or inform infants’ “reasoning” (Baillargeon, 2008) about objects do not, however, shed any light on how such principles might be implemented by the developing neurocognitive system, nor can they be taken to imply that infants are reflectively aware of either the principles that they appear to employ or the inferences that such principles might entail (Fields, 2013a).

Experiments testing visual object tracking or judgments of visual object persistence over short (few second) exposure times, in some cases with brief (few 100 ms) occlusion times, have consistently shown that trajectory information dominates feature information in infants as it does in adults (Treisman, 2006; Scholl, 2007; Flombaum et al., 2008; Fields, 2011b). Young infants do not, however, treat the same trajectories as indicating object persistence that older infants, children, or adults do; 4-month-old infants, for example, are more tolerant of changes in the speeds of objects while occluded (Bremner et al., 2007), and do not recognize occluded bounces as identity-preserving (Bremner et al., 2005). These results suggest both that the excitation of one or more visuo-motor networks that recognize specific curvilinear paths as trajectories is required for the recognition of object persistence during short visual exposures, and that the specificities of such trajectory-recognition networks change over developmental time (Fields, 2011b). As demonstrated by Needham et al. (2005) as well as by many others, however, object features play an increasingly-significant role in both visual object segmentation and re-identification over longer visual exposures, including exposures involving occluders; indeed infants would be incapable of re-identifying objects that had changed positions while unattended—for example, while the infant was sleeping—if this were not the case.

The simplest heuristic with which to re-identify objects across significant gaps in observation extends and operationalizes “Leibniz’s Law” of the identity of indiscernibles: if two objects appear to be the very same thing, then assume, *ceteris paribus* and allowing for “reasonable” changes in location between observations, that they are the very same thing. In a world in which Baillargeon’s (2008) principle of persistence held universally for properties other than location, and in which indiscernible duplicates of objects did not exist, this operationalized version of Leibniz’s Law would be not just a convenient heuristic, but an actual solution to the problem of object re-identification: the same collection of properties would always indicate the same object, and any differences in observed properties would indicate a different object. Even in the world of the infant, however, object properties do sometimes change between observations, and indiscernible duplicates, particularly of manufactured artifacts, do exist. At least by the pre-school years, children supplement Leibniz’s Law with information about the history of an object between perceptual encounters when making judgments about identity. Gutheil et al. (2008), for example, showed that 4 and 5-year olds can employ historical information to distinguish between otherwise-indiscernible artifacts, while both Hood and Bloom (2008) and Frazier and Gelman (2009) showed that 4-year-olds incorporate information about an object’s history into judgments of its value. Such results, together with a broad variety of studies of adult object re-identification (reviewed by Rips et al., 2006; Bullot and Rysiew, 2007; Scholl, 2007; Xu, 2007; Flombaum et al., 2008) and consideration of relevant neuroscience (reviewed by Eichenbaum et al., 2007; Martin, 2007; Bubic et al., 2010; Zimmer and Ecker, 2010), suggest that the incorporation of historical and hence causal information is obligate in human object re-identification (Fields, 2012a). As the histories of objects while they are not observed are typically unknown, the causal histories typically employed in object re-identification across significant gaps in observation are *fictive*: they must be constructed, on demand, whenever an object is re-identified.

Functional neuroimaging studies suggest that the pre-motor action planning system, including areas of inferior (IPL) and superior (SPL) parietal lobules activated by observed actions and motions respectively, is involved in the construction of such fictive causal histories (FCHs; for a review of relevant studies see Fields, 2012a). These parietal areas support perception-action mirroring and are components of the MNS as broadly conceived (e.g., Cattaneo and Rizzolatti, 2009). As discussed in the Section, Action Recognition and Imitation in Infancy above, at least some components of this system appear to be functional from early infancy, and to support imitation in particular. Hence it seems plausible to consider FCH construction as an architecturally-specified function of the action-planning system, including its mirror components, from early infancy onwards. The construction of FCHs can, in other words, be expected to *implement* the “principle of persistence” for objects that remain unobserved for more than a few seconds from infancy onwards.

## Mechanism-driven causation as an obligate analog of agency

As reviewed in the Section, Development of Mechanism-Based Causal Reasoning in Childhood above, children begin to develop an understanding of hidden, internal causes (e.g., batteries) and of non-contact or non-mechanical causes (e.g., remote controls, wires) only during the pre-school years, and only robustly during the late pre-school years. Prior to that, all autonomous or hidden causation—all causation other than observed contact mechanical causation—is attributed exclusively to agents. The construction of FCHs requires the representation of *unobserved* causes; hence the only causes that can be represented by FCHs constructed to re-identify objects prior to the development of an understanding of hidden, non-contact, or non-mechanical causes are agentive causes. *Every* change in location or features of a re-identified object can be expected, therefore, to be associated with some causal agent by the very FCH employed to re-identify that object. If this is correct, such explanations as “someone put it there”, “someone took my toy” or “someone changed the doll’s clothes” are not just typical explanations for change in the infant and early-pre-school worlds, they are the *only possible* explanations for changes. Absent the ability to construct a representation of a causal agent, which may well be an unknown “someone”, change cannot be represented by an FCH and hence is not perceptible: without a causal agent to alter an object’s location or features, the altered object can only be perceived as a *different entity*. It has been suggested, on the basis of this model of object re-identification as involving obligate FCH construction, that an inability to construct agent-driven FCHs and hence an inability to re-identify objects—including other human beings—across gaps in observation may explain the typical social, linguistic, and attentional presentations of autism spectrum disorders (ASDs; Fields, 2012a,c).

If the human bias toward agency is embedded in the very mechanism of object re-identification as proposed here, how can the bias be overridden? How can adults, let alone 4-year-olds, come to realize that inanimate objects can change their own locations, or change their own features, without the intervention of any agent of any kind? How can anyone, for example, come to the realization that hurricanes are not directed toward their destinations by gods? If the model of object re-identification as involving obligate FCH construction is correct, this can only occur if (1) a mechanism represented as contained within or associated with the object itself inferentially replaces the otherwise-obligate external agent in an FCH constructed to re-identify the object; and (2) this mechanism-based FCH out-competes any agent-based FCH as a basis for further inferences about the object. A hurricane must, for example, be represented as containing mechanisms that, by suitable interactions with the hurricane’s environment, move it along a particular trajectory as a self-propelled but inanimate object, and this representation must out-compete representations of the hurricane as moved along its trajectory by some external agent. This can only happen if two conditions are met. First, the pre-motor planning system, and in particular its mirror components, must be able to represent inanimate objects as autonomous causes, at least within particular kinds of contexts, of their own motions and featural changes. Second, either the mechanism-based FCH itself or other representations activated during the course of object re-identification must suppress competing agent-based FCHs. The remainder of this section focuses on the construction of mechanism-based FCHs; the conditions under which such FCHs can override the bias toward agency are considered in the Section, Overriding the Bias Toward Agency.

The function of the MNS within the visuo-motor system is to connect seeing and doing: to re-represent a 3rd person perspective on an action as a 1st person perspective. Typically-developing human beings cannot help but view familiar goal-directed actions such as grasping a coffee cup as intentional when they are performed by other humans, it has been claimed (e.g., Gallese, 2007), because their MNSs map observed actions to goal-oriented intentions. Such inferred intentions may be experienced only as attributions to the observed actor; what is important is that they are available, un- or pre-consciously, for further inferences about the observed agent’s goals or future behavior. It is the failure of this action-to-intention mapping in ASDs—at least for actions without perceptible mechanical consequences—that motivates the “broken mirrors” hypothesis for ASD (Iacoboni and Dapretto, 2006; Oberman and Ramachandran, 2007). The plasticity of the MNS on its input side enables the bias toward agency by enabling the mapping of observed non-biological motions to representations of 1st person actions and their typically-accompanying intentions, and hence the representation of inanimate non-agents as agents (Catmur et al., 2007; Heyes, 2010, 2012). It is this mapping that presumably implements the “irresistible” perception of certain motions as indicative of agency, even if they are executed by animated geometrical shapes (Scholl and Tremoulet, 2000; Scholl and Gao, 2013).

The pre-motor representation of action does not, however, only involve intention; it also, and from the standpoint of executing a planned action, it primarily involves a representation of the directed muscular effort required to successfully complete the action. Directed muscular effort is the intuitive human representation of applied force; the force required to pull or push something is just the directed muscular effort required to pull or push it. The mapping from applied effort to achieved results is learned by actively and experimentally manipulating objects, beginning in early infancy; a correct representation of the applied effort required to execute a motion is what makes imitation, for example, possible. By constructing a pre-motor representation of an observed action, the MNS associates the observed action with a felt sense of applied effort. The actions of other agents, and of other entities interpreted by the MNS as agents, are therefore represented not just as intentional but also as effortful, as transferring effective force. This feeling that force has been transferred is as irresistible as the sense of agency in animations that depict collisions between simple geometric shapes.

In order to plan novel manipulations, the pre-motor system must perform a particular kind of inference: it must predict, on the basis of representations of the object to be manipulated, the motion desired and past experience manipulating similar objects, a representation of the applied force required to achieve the desired motion. To produce an effective motor plan, such force-motion scaling must be *quantitatively* correct. Inferences that preserve relations between the represented components of distinct situations or events are structure mappings (Gentner, 1983); three decades of experimental and computer simulation work have shown that structure mappings are the underlying drivers of analogical inferences (reviewed by Markman and Gentner, 2001; Gentner, 2003; Holyoak, 2005). It has been shown, by an analysis of the mechanisms involved in tool improvisation in both humans and non-human animals, that the force-motion scaling inferences performed by the pre-motor action planning system are structure mappings, and can therefore be considered to be analogies (Fields, 2011a). As visual imagination is implemented by excitations of the same intermediate visual and visuo-motor pathways as visual perception (reviewed by Kosslyn et al., 2006; Moulton and Kosslyn, 2009), any event that can be visualized can be used as input to a structure mapping inference by the human pre-motor action planning system. Functional imaging studies indicate that pre-motor force-motion scaling implements structure-mapping analogies both in abstract but visualizable domains involving forces and motions (Fields, 2012b) and in formal domains representing metaphorical motions (Fields, 2013b).

Structure-mapping analogy provides an inferential mechanism by which hidden, remote, or non-mechanical causes can replace agency as generators of observed actions. Once a situation in which batteries, remote controls, electrical wires, car engines or other such mechanisms has been observed to cause motion or feature changes is available in memory, it becomes available as a base case for analogy; seeing that batteries can make a toy car go, for example, makes it possible to suppose not only that batteries make other things go, but that other inanimate objects somewhat like batteries might make things go. For such analogies to be implemented by the pre-motor planning system as components of FCH construction, their base cases must be represented in a modality that is available to this system, e.g., as a visual imagination. That multiple perceptual and imaginative modalities can excite visuo-motor representations in adults is well-established in the case of tools and their typical uses (reviewed by Lewis, 2006); the results of Loewenstein and Gentner (2005) indicating that exposure to words naming spatial relations facilitates visuo-spatial reasoning in pre-schoolers suggests that such cross-modal connections are available at the developmental stages of interest here. As the construction of an FCH is always the representation of an unobserved causal event, FCH construction always involves structure mapping, even if the mapping is as simple as replacing the initial and final states of an episodic memory of an extended causal event with the initial and final states of an otherwise-unobserved history (Fields, 2012a). The availability of imagined internal or remote mechanisms to pre-motor structure mapping makes them, therefore, available to pre-motor FCH construction. Visuo-motor *imagination* is crucial to this pathway; the incorporation of never-observed internal or remote mechanisms into FCHs would be predicted, on this model, not to occur prior to the age at which such mechanisms could be imagined either visually or verbally. The development of mechanistic reasoning capabilities in the pre-school years, and not before, is consistent with this prediction.

## Overriding the bias toward agency

The considerations reviewed above allow the formulation of a succinct mechanistic model of the bias toward agency. Human infants are born with or rapidly develop abilities to segregate objects from their backgrounds and to re-identify objects as the same individual, persistent things over relatively short gaps in observation. They are also born with or rapidly develop an MNS-mediated ability to represent some observed objects—particularly animate objects such as human beings or other animals—as agents and to represent their actions as intentional or goal-directed. Agents move themselves and alter their own features; even inanimate objects that move themselves and alter their own features are, unless their actions are highly predictable, typically regarded as agents. Early experience moving and altering passive inanimate objects, and observing other people moving and altering passive inanimate objects, permits the construction, by the developing pre-motor system, of FCHs that account for changes in the locations and features of such objects, and hence allows the re-identification of such objects as the same individual things across arbitrarily-long gaps in observation. These FCHs attribute the motions and featural changes of passive inanimate objects to external agents; hence every re-identification of such an object after a gap in observation associates any observed changes in the location or features of that object with some agent, even if that agent is an unknown “someone”. Infants, therefore, have an unavoidable bias towards agency; agency is invoked whenever they re-identify an object.

It is important to emphasize that the invocation of agency required by the above model is not in general conscious. Objects are typically re-identified within the time required for conscious awareness; hence FCH construction is in most cases—and for preverbal infants, arguably in all cases—an un- or pre-conscious cognitive activity. Infants are, therefore, not predicted to be aware of the FCHs they construct, aware of the agents that they represent within these FCHs, or even aware of the process of re-identifying an object. The bias toward agency is, in this model, an automatic, unconscious bias.

Because the bias toward agency is embedded in the process of object re-identification, it is to be expected that it would survive into and through adulthood. What demands explanation, in this model, is not why the bias toward agency survives, but rather how the bias toward agency could ever be overridden. The model is, therefore, consistent with the observation that in some human cultures the bias toward agency is overridden only rarely or in particular circumstances. The model is, in particular, consistent with the ubiquity of culturally-acknowledged supernatural explanations for many phenomena involving changes in the locations or properties of inanimate objects, even ones that might otherwise appear trivial. What is anomalous within the model is the existence of individuals or (sub)cultures in which the behaviors of objects that are not obvious social agents—typically, people or other animals—are routinely explained in terms of non-agentive internal or external mechanisms. The model predicts that such individuals must have had, and such (sub)cultures must widely provide, “discovery” experiences that reveal the workings of previously-hidden mechanisms. It predicts that such individuals must execute particular kinds of inferences—structure mappings—that replace agentive causes with mechanistic causes for both observed and unobserved events. It also predicts that, at least in some cases, the construction of such alternative, mechanism-based FCHs is accompanied by the suppression of competing agent-based FCHs. It predicts, in other words, that mere factual knowledge of mechanistic causes is not enough; such causes must be incorporated into the routine, largely unconscious processing that subserves object re-identification in order to become effective.

The current model is, therefore, *prima facie* inconsistent with the idea that human beings are, in population average, “balanced” between mentalizing and systemizing, as suggested by research carried out with survey instruments such as the empathizing-systemizing quotient (EQ-SQ) developed by Baron-Cohen and colleagues (Baron-Cohen et al., 2003; Goldenfeld et al., 2005; Nettle, 2007). The EQ and SQ instruments do not, however, measure problem solving outcomes, and do not directly measure problem-solving style. Many of the proposed responses, for example “I prefer to read non-fiction than fiction” or “I make it a point of listening to the news each morning” measure activity preferences (Baron-Cohen et al., 2003, p. 368). While the relatively high SQ scores of scientists, technologists, engineers and mathematicians tested (Baron-Cohen, 2008) suggest that the SQ instrument indirectly measures systemizing ability, similar results correlating EQ score with empathizing ability, in the strict sense of empathizing *accuracy*, are not available. Very high EQ scores have, indeed, been shown in some cases to correlate with self-reports of symptomatic psychosis (Brosnan et al., 2010), while very high SQ scores correlate with ASD. The “balance” between EQ and SQ scores in the general population may, therefore, reflect a survey design that emphasizes personality and social-interaction characteristics, not actual problem-solving strategies. Many “balanced” individuals may nonetheless be biased toward attributions of agency, particularly when under stress or faced with unanticipated or extreme events. The results of Kelemen et al. (2013) indicate, moreover, that while the bias toward agency can be overridden, it is not extinguished even in professional physical scientists, individuals who would be expected to have very high SQ.

The current model predicts that individuals can override the bias toward agency, and hence develop systemizing skills including the ability to discover previously-hidden causal mechanisms, only if they have both appropriate learning experiences and well-developed structure-mapping abilities. Mere exposure to the behavior of inanimate mechanisms, or even to hidden parts such as batteries that have causal power, is unlikely to be sufficient for learning; as is often emphasized in relation to classroom learning (e.g., Pintrich et al., 1993), a rich environment in which both complex behaviors executed by mechanisms and the parts causally responsible for such behaviors are made both observationally and motivationally salient can be expected to be necessary. The SQ instrument arguably measures the extent to which causal mechanisms are salient to an individual; high SQ scores can, therefore, be expected to be predictive of an enhanced ability to *learn* systemizing skills. While structure-mapping or analogical reasoning ability is widely acknowledged as central to general intelligence (e.g., Gentner, 2003) and is typically measured as a component of general intelligence, specific tests of structure-mapping ability, especially for use in young children, have yet to be developed.

The model of the bias toward agency that is proposed here can be tested on a number of different levels. It is not clear, for example, how young children categorize objects such as batteries, remote controls or wires; were it shown that children routinely categorized such objects as agents, the present model would require revision. A version of the EQ-SQ instrument designed for evaluating children as young as 4 years has been developed (Auyeung et al., 2009). The current model would predict a positive correlation between childhood SQ, visual imaginative ability, and performance on tasks probing understanding of mechanisms such as those employed by Sobel et al. (2007) or Sobel and Buchanan (2009) with pre-schoolers. A positive correlation is similarly predicted between any of the above measures and a focused measure of pre-school analogical reasoning ability. A finding of no or negative correlations between any of these measures would cast doubt on the central mechanistic claim of the model, the claim that attributions of mechanism-based causation require imagination-dependent structure mapping.

A second sensitive test would be achieved by combining the experimental designs of Gutheil et al. (2008) and Hood and Bloom (2008). Both designs require pre-school subjects to re-identify one of two identically-featured objects. In the design of Gutheil et al. (2008), a human experimenter carries one of the objects out of the room and then back in; the subject’s task is to recognize that this agent-executed causal process preserves object identity. In the design of Hood and Bloom (2008), the experimenter is replaced by a “copying machine” into which one of the objects is put; in this case, the subject’s task is to distinguish the original object from the “copy”. Interleaving actions on the two objects by the experimenter and by the machine would test subject’s abilities to construct FCHs that incorporate both mechanisms and agents as either preservers or disruptors of object identity. Such a test would be rendered more stringent by adding an unobserved process, carried out either by the experimenter or the machine, that produced featural changes to the object meant to be recognized as “the same thing”. Performance on these re-identification tests would be expected to correlate with childhood SQ score as discussed above.

Direct tests of pre-motor involvement in object re-identification are complicated by the commonly-observed activation of the pre-motor system during episodic recall (Moscovitch, 2008; Ranganath, 2010) and task-specific attention (Cabeza et al., 2008; Uncapher and Wagner, 2009; Ranganath, 2010). However, consistent activation of SPL over IPL during object re-identifications requiring mechanism-driven causation and of IPL over SPL during object re-identifications requiring agency-driven causation would lend credence to the model (Fields, 2012a). Correlation of higher activation of SPL with higher SQ, and of higher IPL activation with higher EQ, would also tend to confirm the model. As the model relies on the attribution of agency being a default strategy implemented by the pre-motor system, further tests of this assumption would test the model. Targeted suppression of activity in temporal-parietal junction (TPJ) by transcranial magnetic stimulation (TMS), for example, would be expected to bias subjects in favor of mechanistic models of causation. Specific suppression of the “wolfpack effect” (Scholl and Gao, 2013) by TMS to TPJ, for example, would tend to confirm the model. A replication of the explanation-validity judgment test of Kelemen et al. (2013) using either fMRI, where enhanced IPL over SPL activation would be expected on time-limited trials, or TMS to TPJ would also test the model.

## Conclusion

The developmental and neurocognitive considerations discussed here suggest that the human bias toward agency is encoded in the pre-motor mechanisms that enable object re-identification across gaps in observation, and that overriding it requires the construction, again by the pre-motor system, of structure-mapping analogies that replace agents with mechanisms as generators of action. Whether this human mechanism for object re-identification is a result of selective pressure for a “social brain” is unknown; both studies of MNS function (Nassi and Callaway, 2009) and studies of object re-identification (Munakata et al., 2001; Flombaum et al., 2008) in non-human primates indicate broad similarities with human capabilities. It is possible, however, that the development of agentive reasoning and hence of social brains is itself driven, at least in part, by selective advantages conferred by the ability to re-identify objects as individuals. While memory for places has been extensively studied even in non-primates (e.g., Gould et al., 2010) and many animals are known to mate for life, little in general is known about the evolutionary origins of memory for individual objects. Specific studies of the re-identification of inanimate objects, such as stones or other items used as tools, by both non-human primates and other animals would contribute to understanding the evolutionary history of human object re-identification capabilities. Studies addressing the ability of non-human animals to re-identify individual objects after hidden manipulations of their locations or salient features would be particularly revealing.

On a more general note, the cultural history of the human species, particularly over the past 500 years, is a history of progressive challenges to the assumption, whether implicit and architectural or explicit and cultural, that all autonomous causation involves agency. The bias toward agency is clearly economically significant in cultures heavily invested in, and dependent upon, technological capabilities. It is also politically significant, as demonstrated not only by the historic and contemporary roles of religion in politics, but also by studies linking low SQ to reasoning deficits such as “jumping to conclusions” (Brosnan et al., 2013). Understanding the neurocognitive mechanisms underlying the bias toward agency, its maintenance in the face of experience, and the experiential and cognitive requirements for overriding it may, therefore, prove to be of broad significance for human culture.

## Conflict of interest statement

The author declares that the research was conducted in the absence of any commercial or financial relationships that could be construed as a potential conflict of interest.
